# The associations of muscle-strengthening exercise with recurrence and mortality among breast cancer survivors: a systematic review

**DOI:** 10.1186/s12966-024-01644-0

**Published:** 2024-09-10

**Authors:** Oliver W.A. Wilson, Kaitlyn M. Wojcik, Dalya Kamil, Jessica Gorzelitz, Gisela Butera, Charles E. Matthews, Jinani Jayasekera

**Affiliations:** 1 Health Equity and Decision Sciences Research Laboratory, Division of Intramural Research, National Institute of on Minority Health and Health Disparities, National Institutes of Health, Bethesda, MD 20892 USA; 2 Department of Health and Human Physiology, University of Iowa, Iowa City, IA USA; 3 Office of Research Services, National Institutes of Health Library, Bethesda, MD USA; 4 Metabolic Epidemiology Branch, National Cancer Institute, National Institutes of Health, Bethesda, MD USA

**Keywords:** Exercise, Physical activity, Resistance training, Weight lifting, Breast cancer, Survival, Survivorship, Recurrence, Mortality, Death

## Abstract

**Background:**

Our systematic review aimed to critically evaluate empirical literature describing the association of muscle-strengthening exercise (MSE) with recurrence and/or mortality among breast cancer survivors.

**Methods:**

We included English-language empirical research studies examining the association between MSE and recurrence and/or mortality among females diagnosed with breast cancer. Seven databases (MEDLINE, PsycINFO, Embase, Scopus, Web of Science, Cochrane CENTRAL, and CINAHL) were searched in September 2023. Quality was appraised using the Mixed Methods Appraisal Tool. Results are summarized descriptively.

**Results:**

Five sources were identified. MSE measurement differed in relation to the description of the MSE (i.e., muscle-strengthening vs. strength training), examples of activities (e.g., sit-ups or push-ups vs. calisthenics vs. circuit training), and exercise frequency (i.e., days vs. times/week). Findings offer provisional evidence that some MSE may lower the hazards of recurrence and mortality. This association may vary by race, weight status, and menopausal status.

**Conclusions:**

In summary, limited available evidence suggests that MSE may lower the hazards of recurrence and mortality. More consistent measurement and analyses would help generate findings that are more readily comparable and applicable to inform clinical practice. Further research is needed to improve understanding of the strength and differences of these associations among underserved and underrepresented women.

**Supplementary Information:**

The online version contains supplementary material available at 10.1186/s12966-024-01644-0.

## Background

Breast cancer is the most commonly diagnosed cancer among women, [1] and among the most costly types of cancer to treat [2]. For instance, one in eight women in the U.S. are diagnosed with breast cancer every year, and there are currently over four million breast cancer survivors in the U.S. [3] The incidence of breast cancer is projected to increase by more than 50% by the year 2050 [4]. However, due to prolonged treatment, breast cancer survivors may experience reduced muscular strength, mobility, and daily activity [5, 6].

Muscle-strengthening exercise (MSE; i.e., resistance / strength / weight training or exercise) can improve short-term outcomes such as anxiety, depression, fatigue, health-related quality of life, lymphedema, and physical function among breast cancer survivors [7–10]. Therefore, the long-term benefits of MSE on events such as recurrence and mortality may be influenced by improvements in short-term outcomes associated with MSE. Moreover, MSE may also offer benefits relating to recurrence and mortality via the regulation of hormones and cytokines (myokines), as well as changes in body composition resulting in improvements in metabolic regulation and immunity among breast cancer survivors [11–13]. As a result, guidelines endorsed by the American Cancer Society (ACS), American College of Sports Medicine (ACSM), and Centers for Disease Control recommend that cancer survivors engage in MSE at least two times per week in addition to aerobic exercise [7]. It is also worth noting that these guidelines are largely based on evidence from trials involving early-stage breast cancer survivors, and that the guidelines do not yet refer to benefits of MSE in relation to recurrence or mortality.

MSE was introduced to U.S. [14] and international [15] guidelines approximately 15–25 years ago. For breast cancer survivors specifically, fewer studies have been designed or published relating to the long-term benefits of MSE in comparison to aerobic exercise, with no review, let alone a meta-analysis, to date. In contrast, over the past five years alone, the association of aerobic exercise with hazards of recurrence and mortality among breast cancer survivors has been the focus of five systematic reviews/meta-analyses encompassing more than 40 articles [16–20]. Thus, compared to aerobic exercise research, research on the association of MSE with recurrence and/or mortality among breast cancer survivors is in its infancy.

In summary, MSE promotion tends to be overlooked in clinical settings, [21] with only 16.5% of breast cancer survivors in the U.S. currently meeting national MSE guidelines compared to 35.7% meeting aerobic guidelines [22]. Thus, the overarching goal of this systematic review was to collate and critically evaluate empirical literature describing the association of MSE with recurrence and/or mortality to support the promotion of MSE among breast cancer survivors in clinical settings.

## Methods

We conducted a systematic review following the established Preferred Reporting Items for Systematic reviews and Meta-Analyses (PRISMA) guidelines (See Supplement 1 for the PRISMA Checklist) [23, 24]. Synthesis and presentation of findings was based on Cochrane methods [25]. The study was registered in Open Science Framework (doi: 10.17605/OSF.IO/NQRY8).

### Eligibility criteria

Our review included English-language empirical research studies examining the independent association between MSE and recurrence and/or mortality among females diagnosed with breast cancer. Inclusion and exclusion criteria are detailed in Table 1 below. In the absence of a prior review, we extended our search back two decades to include the period before and after the introduction of MSE into exercise guidelines in the late 2000s and early 2010s [14, 15].

**Table 1:** Inclusion and exclusion criteria

Criteria	Inclusion	Exclusion
Date	• Published 2003 to present	• Published prior to 2003
Population	• Breast cancer survivors	• Non-human subjects • Non-breast cancer survivors
Exercise	• Measure MSE (including resistance training, strength training, weight training, and weightlifting)	• Do not measure MSE (including resistance training, strength training, weight training, and weightlifting) or combine MSE with aerobic exercise participation for analyses
Concept	• Studies that include breast cancer recurrence, secondary cancer, breast cancer mortality, mortality events	• The absence of outcomes stated within inclusion criteria
Type of Evidence	• Primary empirical research studies available in full text	• Reviews and meta-analyses • Editorials (e.g., perspectives, commentaries) • Abstracts, proceedings, or posters • Dissertations/theses • Research protocols • Case reports • Patents • Articles for which the full text cannot be obtained
Language	• English	• Not available in English

Notes. MSE = Muscle-strengthening exercise

### Information sources

The search strategy was developed by a trained librarian (GB) and included a combination of terms relating to breast cancer, survivorship, exercise (physical activity), recurrence, and mortality. The current review reports on studies that focused on MSE, which were separated from those that focused exclusively on participation in aerobic physical activity. The search strategy was developed using an iterative approach with three rounds by a trained librarian (GB). Each preliminary search was performed in the PubMed/MEDLINE database constrained to research published from January 2003 until September 2023 with English-only language restrictions. The search strategy included a combination of keywords, synonyms, Medical Subject Headings (MeSH) terms, and Emtree terms. The search was performed across the following seven widely used scientific databases: MEDLINE via PubMed (National Library of Medicine), PsycINFO (American Psychological Association), Embase (Elsevier), Scopus (Elsevier), Web of Science Core Collection (Clarivate Analytics), Cochrane CENTRAL (Wiley & Sons), and CINAHL (EBSCO). The date of our search was September 18, 2023. OW and KW performed a pilot test of the preliminary PubMed/MEDLINE search strategy results using Covidence [26]. A sub-sample of those included after full text screening were used to pilot the data extraction tool by OW and KW. Search strategies for each information source are reported in Supplement 2.

### Selection process

Database records were imported into EndNote x20 (Clarivate) reference manager software where duplicates were removed. Covidence screening software (Veritas Health Innovation, Melbourne, Australia) was used to further identify duplicates and perform screening eligibility of records [26]. Three co-authors (OW, DK, KW) independently screened both title/abstracts and discrepancies were resolved via discussion.

### Data collection process

Data were extracted into Excel to ensure consistency and inter-reviewer reliability. Two authors (KW, OW) extracted information on the study location, study purpose, research design, target population and setting, start and end date, number of participants, data source, statistical analysis technique, demographic information, breast cancer treatment and tumor characteristics, exercise measurement methods, social determinants of health, measure(s) of association, main results, study limitations, funding source, and conflicts of interest. Authors were contacted to attempt to acquire missing information.

### Quality appraisal

Article quality was appraised using the Mixed Methods Appraisal Tool (MMAT) [27]. The MMAT includes two screening questions and five items to appraise the quality of quantitative non-randomized research. Items pertain to sample representativeness, measure appropriateness, data completeness, whether confounders are accounted for in design or analysis, and whether changes in the exposure were considered. Each item is appraised as either “yes”, “no”, or “can not tell”. We set the threshold for data completeness at 80% [28]. We used only items 3.1–3.4 from the quantitative non-randomized criteria; additionally, for items 3.2 and 3.4, we focused on the methodology used to assess the association of MSE with recurrence and/or mortality (Table 3).

### Effect measures

Where possible, hazard ratios were extracted regarding the association of MSE with recurrence and/or mortality.

### Synthesis methods

Due to the heterogeneity in MSE measurement and analytical methods used in published studies, we were unable to summarize the results in a meta-analysis. Therefore, we summarized the study findings using a narrative approach as outlined by Cochrane [25]. Accordingly, we summarized the study characteristics, quality appraisal, muscle-strengthening exercise measurements, guidelines, and MSE effect estimates in a narrative synthesis with accompanying tables offering extra details. This review included a review of published articles and study-level results, and therefore did not require submission for institutional review board approval or exemption.

### Certainty

According to Cochrane, narrative approaches to synthesizing results based on statistical significance are severely limited [25]. Our approach to interpreting data is based on an evaluation of the magnitude, direction, and precision of the effect estimates rather than binary significance testing [29]. This approach allowed us to evaluate the clinical relevance of the results in the absence of established minimally clinically important differences for MSE or the reporting of absolute differences in the published studies. Since evidence falls on a continuum, dichotomization into significant vs. non-significant would devalue the information available in the data reported [30, 31].

## Results

### Study selection

**Fig. 1:**
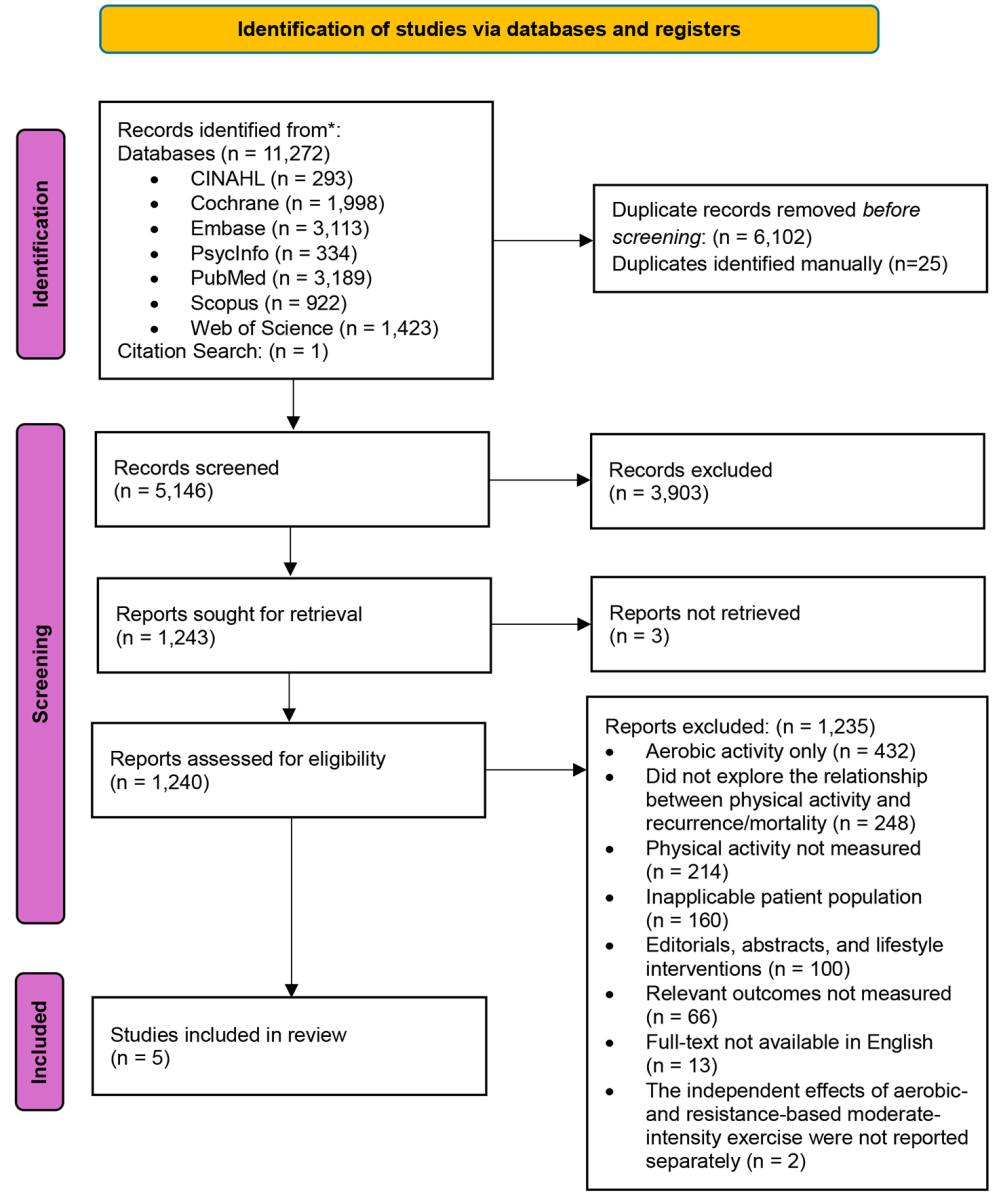
Article identification process using PRISMA research framework [24]

Initial searches retrieved 5,146 sources after removing duplicates. These were screened at the title and abstract levels, followed by a full-text review of the 1,240 remaining sources. We were unable to retrieve full-text copies of three sources via Inter Library Loans or by contacting authors directly. Five articles were identified and proceeded to extraction (Fig. 1). Two studies that included MSE as a part of exercise interventions were excluded, as neither reported on the independent effects of MSE on recurrence and/or mortality [32, 33].

### Study characteristics

Characteristics describing each article are reported in Table 2. The articles analyzed data collected in the U.S. (*N* (no. of articles) = 4), [34–37] and Canada (*N* = 1) [38]. The review included cross-sectional (*N* = 2) [34, 38], prospective (*N* = 2), [35, 37] and retrospective cohort (*N* = 1) analyses [36]. All articles focused on female breast cancer survivors, and all samples were predominantly White apart from one that focused exclusively on African American women [34]. Most participants also had a favorable socioeconomic status (See Table 2).

**Table 2:** Study characteristics

Author	Location (Data source)	Design	Participant characteristics
Ansa et al., 2015 [34]	U.S. - Florida (Morehouse School of Medicine)	Cross-sectional (*n* = 191)	Age: >18 years; M = 56.3 ± 11.4 years Gender: 100% female Race: 100% African American SES: 78.7% ≥ college education; 32.1% annual income ≥$50,000 Disease stage: 41% I; 27.0% II, 21.6% III or IV, 10.3% don’t know Time since diagnosis: Not reported Treatment stage: ≥1-year post-treatment *English speaking
Forbes et al., 2015 [38]	Canada (Nova Scotia Cancer Registry)	Cross-sectional (*n* = 248)	Age: 18–80 years Gender: 100% female Race: 97% White; 3% Other SES: 45% ≤ high school; 55% postsecondary; 66% <60,000; 34% ≥60,000; 66% employed; 34% not employed Disease stage: 59% I; 32% II; 9% III/IV Time since diagnosis: mean: 4.3 years; 65% < 5 years; 35% ≥ 5 years Treatment: 98% surgery; 65% radiation; 47% chemotherapy; 51% hormonal therapy; 24% currently receiving treatment *Residents of Nova Scotia
Fortner et al., 2023 [35]	U.S. - National (Nurses’ Health Studies)	Prospective cohort (*n* = 9308)	Age: NHSI: M = 71.3 (SD = 7.2); NHSII: M = 53.3 (SD = 5.3) Gender: NHSI + NHSII: 100% female Race: NHSI: 1% Asian, 1% Black, 95% White, 3% Unknown/multi-racial; NHSII: 2% Asian, 1% Black, 94% White, 3% Unknown/multi-racial SES: ~20% in each neighborhood SES quintile Disease stage: NHSI: 66% I, 28% II, 6% III; NHSII: 60% I, 33% II, 8% III; Time since diagnosis: Mean 26.5 months Treatment: NHSI: 48% hormone therapy only, 7% chemotherapy only, hormone and chemotherapy 18%; NHSII: 31% hormone therapy only, 17% chemotherapy only, hormone and chemotherapy 40% *≥1 year post-diagnosis
Tarasenko et al., 2018 [36]	U.S. - National (NHIS Linked Mortality Files)	Retrospective cohort (*n* = 2885)	Age: ≥18 years Gender: 100% female Race: Predominantly white SES: Not reported Disease stage: Not collected Time since diagnosis: ≥3 years Treatment: Not collected *excluding non-melanoma skin cancer, unknown age at diagnosis, those who died of accidents, those who were diagnosed and died in the same year
Troeschel et al., 2023 [37]	U.S. - California (Pathways)	Prospective cohort (*n* = 1964 (mortality); *n* = 1,924 recurrence)	Age at diagnosis: 61.2 ± 11.5 years Gender: 100% female Race: 71.4% White SES: 54.2% ≥ college education; 52.6% ≥$70k Disease stage: 57.9% Stage I, 32.7% Stage II, 8.8% Stage III, 0.6% Stage IV Time since diagnosis: shortly after Treatment: 96.8% any surgery; 47.5% any chemotherapy or other anti-cancer drugs; 43.7% any radiation therapy; 73.9% any hormonal therapy; 99.8% any treatment (any of the above)

Notes. BC = Breast cancer BCS = Breast cancer survivors; CS = Cancer survivors; NHIS = National Health Interview Survey; NHS = Nurses’ Health Study; SES = Socio-economic status

### Quality appraisal

All articles had a clear research question, collected appropriate data to answer research questions, included participants that were representative of the target population, and had complete data. All but one article [34] measured MSE in a way that the proportion of participants meeting MSE guidelines could be determined, and three articles [35–37] accounted for confounders in the design and/or analysis (Table 3).

**Table 3:** Quality appraisal

Author	Data Collection Method	S1	S2	3.1	3.2	3.3	3.4
Ansa et al., 2015 [34]	Survey, results from lifestyle assessment tool, focus group discussion	✓	✓	✓	X	✓	X
Forbes et al., 2015 [38]	Survey	✓	✓	✓	✓	✓	X
Fortner et al. 2023 [35]	Medical chart review, survey	✓	✓	✓	✓	✓	✓
Tarasenko et al., 2018 [36]	Survey	✓	✓	✓	✓	✓	✓
Troeschel et al., 2023 [37]	Survey	✓	✓	✓	✓	✓	✓

Notes. S1: Are research question(s) clear?; S2: Is data collection appropriate to answer research question(s)?; 3.1: Are participants representative of target population?; 3.2: Are measurements appropriate for the outcome (recurrence/mortality) and exposure (muscle-strengthening exercise participation)?; 3.3: Is outcome data complete?; 3.4 Are confounders (e.g. individual, clinical, contextual characteristics) accounted for in the design and/or analyses?; ✓= Yes; X = No; ? = Can not tell

### Muscle-strengthening exercise measurement and guidelines

The stated or implied wording of MSE measures differed across articles, where three used muscle-strengthening, [34, 36, 38] and two used strength training [35, 37]. All four articles that included an example of activities mentioned weightlifting or lifting weights, [35–38] with other exercise examples including sit-ups or push-ups, [38] calisthenics, [36] and circuit training [37]. Four articles measured participation frequency, [34, 36–38] two measured duration, [35, 38] and one measured exercise type [38]. Various guidelines (e.g., American College of Sports Medicine) [14, 39–41] were stated or implied in three articles [36–38]. All studies also examined aerobic exercise. However, only one article reported analyses regarding the association between MSE and mortality while adjusting for aerobic exercise participation (Table 4).

**Table 4:** Muscle-strengthening exercise measurement and guidelines

Author	Characteristics Measured	Guidelines (Source)	Proportion meeting guidelines
Ansa et al., 2015 [34]	Muscle strengthening (undefined) in past month (yes/no)	-	-
Forbes et al., 2015 [38]	Muscle-strengthening activity days/week (e.g. weightlifting, sit-ups, or push-ups); duration (min/session), Type of activity [42]	≥ 2 days/week for ≥ 30 min/session (ACSM, USDHSS) [14, 39, 40]	23.0%
Fortner et al. 2023 [35]	Strength training (weight training and/or resistance exercise with arm and/or leg weights) duration/week	-	-
Tarasenko et al., 2018 [36]	Muscle-strengthening activity (e.g. physical activities specifically designed to STRENGTHEN muscles such as lifting weights or doing calisthenics) days/week	≥ 2 days/week (ACSM, ACS) [39, 40]	13.2%
Troeschel et al., 2023 [37]	Strength training (e.g. weight lifting, free weights, circuit training) exercise times/week	> 2 times/week (ACS/ ASCO) [41]	22.9%

Notes. ACS = American Cancer Society; ACSM = American College of Sports Medicine; ASCO = American Society of Clinical Oncology; USDHHS = U.S. Department of Health and Human Services

### Association of muscle-strengthening exercise with recurrence and mortality (Table 5)

#### Recurrence (*N* = 3)

Troeschel et al. [37] suggest that some MSE (1–2 times/wk) was associated with lower hazards of recurrence compared to none (Hazard Ratio [HR] = 0.81 (95% Confidence Interval [CI]: 0.50, 1.29)), and that more MSE (> 2 times/wk) was associated with higher hazards of recurrence compared to none (HR = 1.26 (0.84, 1.90)). However, in both instances, the data were consistent with parameter values ranging from considerably lower to considerably to considerably higher hazards of recurrence. A small (*n* = 248) descriptive cross-sectional study found that a higher proportion of women who were recurrence free (24%, *n* = 57) met MSE guidelines compared to those with recurrence (9%, *n* = 1) [38], whereas another small (*n* = 191) cross-sectional study of African American women found no apparent association between MSE and recurrence [34].

#### Breast cancer-specific mortality (*N* = 3)

Fortner et al. [35] suggest that some MSE (> 0–1 MET-hrs/wk) was associated with lower hazards of breast cancer-specific mortality compared to none (HR = 0.59 (0.33, 1.05)), and that more MSE (≥ 1 MET-hrs/wk) may be associated with even lower hazards (HR = 0.51 (0.25, 1.02)). These associations were evident even after adjusting for pre-diagnosis MSE. Troeschel et al. [37] reported that some MSE (1–2 times/wk) was associated with lower hazards of breast cancer-specific mortality compared to none (HR = 0.26 (0.08, 0.86)). Unlike Fortner et al., Troeschel et al. [37] reported that more MSE (> 2 times/wk) does not offer appear to offer additional benefits (HR = 0.84 (0.40, 1.76)), with data consistent with parameter values ranging from considerably lower to considerably high hazards of breast cancer-specific mortality. Tarasenko et al. [36] reported no association between meeting MSE guidelines and breast cancer-specific mortality (HR = 0.94 (0.59, 1.49)), but data were consistent with parameter values ranging from considerably lower to considerably higher hazards of breast cancer-specific mortality.

#### Cardiovascular disease specific mortality (*N* = 1)

Tarasenko et al. [36] reported that those who met MSE guidelines (HR = 0.69 (0.36, 1.33)) had lower cardiovascular disease specific mortality compared to those who did not. Parameter values ranged from considerably lower to higher hazards of cardiovascular disease specific mortality.

#### All-cause mortality (*N* = 3)

Fortner et al. [35] reported that some MSE (> 0–1 MET-hrs/wk) was associated with lower hazards of all-cause mortality (HR = 0.89 (0.64, 1.25)), though data were consistent with parameter values ranging from lower to higher hazards of all-cause mortality. Fortner et al. [35] also reported that more MSE (≥ 1 0 MET-hrs/wk) may offer additional benefits compared to none (HR = 0.61 (0.39, 0.97)). These associations were evident even after adjusting for pre-diagnosis MSE. Troeschel et al. [37] reported that some MSE (1–2 times/wk) was associated with lower hazards of all-cause mortality compared to none (HR = 0.63 (0.41, 0.98)). However, Troeschel et al. also reported that more MSE (> 2 times/wk) may not offer any additional benefits (HR = 0.99 (0.69, 1.42)). Data were consistent with parameter values ranging from lower to higher hazards of all-specific mortality. Tarasenko et al. [36] reported no association between meeting MSE guidelines and all-specific mortality (HR = 0.91 (0.66, 1.26)), but data were consistent with parameter values ranging from lower to higher hazards of all-cause mortality.

#### Variation based on individual and clinical characteristics

Only Ansa et al., [34] who focused on African American women, examined a sample that was not predominantly White [34]. Sub-group analyses based on BMI reported by Fortner et al. [35] suggest that those with a BMI < 25 kg/m^2^ only see a reduction in all-cause mortality when participating in high-levels of MSE, whereas some MSE may benefit those with a BMI ≥ 25 kg/m^2^ and additional MSE offers further benefits. Fortner et al. also reported slightly higher benefits regarding reduced hazards of breast cancer-specific mortality in postmenopausal women compared to the overall sample.

**Table 5:** Findings on the association of muscle-strengthening exercise with recurrence and mortality among breast cancer survivors

Author	Findings
	**Recurrence**
Ansa et al., 2015 [34]	The proportion of those participating in muscle strengthening in the past month was examined among those with breast cancer recurrence and those free of breast cancer recurrence. Results are reported below: • Among those with breast cancer recurrence, 69.7% (*n* = 23) reported muscle strengthening in the past month vs. 30.3% (*n* = 10) who did not • Among those free of breast cancer recurrence, 67.6% (*n* = 94) reported muscle strengthening in the past month vs. 32.4% (*n* = 45) who did not • *p* = 0.82 *Variables adjusted for: None
Forbes et al., 2015 [38]	The proportion of those who met strength exercise guidelines was compared between those with and without recurrence. Results are reported below: • 9% (*n* = 1) with recurrence met guidelines • 24 (*n* = 57) of those who were recurrence free met guidelines • *p* = 0.430 *Variables adjusted for: None
Troeschel et al., 2023 [37]	The association of different levels strength training with breast cancer recurrence was examined by computing Cox proportional hazard ratios. Results are reported below: • Referent: None • 1–2 times/wk: HR = 0.81 (95% CI: 0.50, 1.29) • > 2 times/wk: HR = 1.26 (95% CI: 0.84, 1.90) *Variables adjusted for: Age, tumor stage, ER status, chemotherapy, Herceptin, radiotherapy, comorbidities, smoking status, income, and education level, diet, alcohol, aerobic exercise, sedentary behavior, body weight
	**Breast cancer-specific mortality**
Fortner et al. 2023 [35]	The association of different levels of strength training (measured in MET-hrs/wk) with breast-cancer specific mortality was examined by computing Cox proportional hazard ratios. Results are reported below: • Referent: 0 MET-hrs/wk • > 0–1 MET-hrs/wk: HR = 0.59 (95% CI: 0.33, 1.05) (Model 1); HR = 0.60 (95% CI: 0.33, 1.08) (Model 2) • ≥ 1 MET-hrs/wk: HR = 0.51 (95% CI: 0.25, 1.02) (Model 1); HR = 0.49 (95% CI: 0.23, 1.06) (Model 2) *Variables adjusted for: (Model 1) estrogen receptor (ER)/progesterone receptor (PR) status, treatment with tamoxifen, aromatase inhibitor, Herceptin, and/or chemotherapy, stage at diagnosis (I-III), pre-diagnosis hormone therapy use, pre-diagnosis BMI, BMI change from pre-diagnosis to current, alcohol consumption, smoking status, aspirin use, and neighborhood SES.; (Model 2) same variables as Model 1 + pre-diagnosis exercise **Results for postmenopausal women only, and stratified by BMI are reported in Supplementary Tables 5 and 6 respectively of Fortner et al. 2023 [35]
Tarasenko et al., 2018 [36]	The association of meeting muscle-strengthening exercise guidelines and breast cancer-specific mortality was examined by computing Cox proportional hazard ratios. Results are reported below: • Referent: <2 times/wk • ≥ 2 times/wk: HR = 0.94 (95% CI: 0.59, 1.49) *Variables adjusted for: Age (continuous), race/ethnicity, education level, marital status, insurance status, self-rated health, activity limitations, smoking status, BMI categories, number of comorbid conditions, and age at first cancer diagnosis
Troeschel et al., 2023 [37]	The association of different levels strength training with breast-cancer specific mortality was examined by computing Cox proportional hazard ratios. Results are reported below: • Referent: None • 1–2 times/wk: HR = 0.26 (95% CI: 0.08, 0.86) • > 2 times/wk: HR = 0.84 (95% CI: 0.40, 1.76) *Variables adjusted for: Age, tumor stage, ER status, chemotherapy, Herceptin, radiotherapy, comorbidities, smoking status, income, and education level, diet, alcohol, aerobic activity, sedentary behavior, body weight
	**Cardiovascular disease specific mortality**
Tarasenko et al., 2018 [36]	The association of meeting muscle-strengthening exercise guidelines and cardiovascular disease specific mortality was examined by computing Cox proportional hazard ratios. Results are reported below: • Referent: <2 times/wk • ≥ 2 times/wk: HR = 0.69 (95% CI: 0.36, 1.33) *Variables adjusted for: Age (continuous), race/ethnicity, education level, marital status, insurance status, self-rated health, activity limitations, smoking status, BMI categories, number of comorbid conditions, and age at first cancer diagnosis
	**All-cause mortality**
Fortner et al. 2023 [35]	The association of different levels of strength training (measured in MET-hrs/week) with all-cause mortality was examined by computing Cox proportional hazard ratios. Results are reported below: • Referent: 0 MET-hrs/wk • > 0–1 MET-hrs/wk: HR = 0.89 (95% CI: 0.64, 1.25) (Model 1); HR = 0.90 (95% CI: 0.64, 1.27) (Model 2) • ≥ 1 MET-hrs/wk: HR = 0.61 (95% CI: 0.39, 0.97) (Model 1); HR = 0.65 (95% CI: 0.40, 1.06) (Model 2) *Variables adjusted for: (Model 1) ER/PR status, treatment with tamoxifen, aromatase inhibitor, Herceptin, and/or chemotherapy, stage at diagnosis (I-III), pre-diagnosis hormone therapy use, pre-diagnosis BMI, BMI change from pre-diagnosis to current, alcohol consumption, smoking status, aspirin use, and neighborhood socio-economic status.; (Model 2) same variables as Model 1 + pre-diagnosis physical activity **Results for postmenopausal women only are reported in Supplementary Table 5 of Fortner et al. 2023 [35]
Tarasenko et al., 2018 [36]	The association of meeting muscle-strengthening exercise guidelines and all-cause mortality was examined by computing Cox proportional hazard ratios. Results are reported below: • Referent: <2 times/wk • ≥2 times/wk: HR = 0.91 (0.66, 1.26) *Variables adjusted for: Age (continuous), race/ethnicity, education level, marital status, insurance status, self-rated health, activity limitations, smoking status, BMI categories, number of comorbid conditions, and age at first cancer diagnosis
Troeschel et al., 2023 [37]	The association of different levels strength training with all-cause mortality was examined by computing cox proportional hazard ratios. Results are reported below: • Referent: None • 1–2 times/wk: HR = 0.63 (95% CI: 0.41, 0.98) • > 2 times/wk: HR = 0.99 (95% CI: 0.69, 1.42) *Variables adjusted for: Age, tumor stage, ER status, chemotherapy, Herceptin, radiotherapy, comorbidities, smoking status, income, and education level, diet, alcohol, aerobic activity, sedentary behavior, body weight

Notes. BMI = Body mass index; ER = estrogen receptor; HR = Hazard ratio; MET = Metabolic equivalent; PR = progesterone receptor; CI = Confidence Interval

## Discussion

Findings of our review of the relatively limited available literature (*N* = 5 studies) tentatively suggest that MSE may lower the hazards of recurrence and mortality among breast cancer survivors [35–38]. This aligns with the findings regarding MSE in prospective research involving survivors of other cancers in relation to mortality [43]. However, evidence is mixed as to whether additional MSE offered benefits in reducing the hazards of mortality [35, 37]. MSE measurement differed across articles in relation to the description of the MSE (i.e., muscle-strengthening vs. strength training), examples of activities (e.g., sit-ups or push-ups vs. calisthenics vs. circuit training) and exercise frequency (i.e., days vs. times per week). These differences, combined with differences in the categorization of MSE participation and therefore comparison groups within analyses, presented challenges comparing results across articles and prevented formal meta-analysis in the current review.

Our review has several limitations. Our exclusion criteria required articles that examined associations broadly among cancer survivors, and any articles that combined MSE with aerobic exercise into a single exercise variable were excluded. We found no studies on clinical trials evaluating the independent effects of MSE on recurrence and/or mortality among breast cancer survivors. This finding is consistent with a recent systematic review of MSE trials among breast cancer survivors that included 41 articles that reported more than 30 potential benefits of MSE, however, none of these benefits included changes in the risk or hazards of recurrence or mortality [10]. The lack of trial data on the long-term effects of MSE may be explained by the lack of adequate long-term follow up, sample size, and/or power in clinical trials. Over the last two decades, the prognosis of early-stage breast cancer has improved remarkably due to recent advances in breast cancer treatment [1]. For instance, studies report that up to 99% of women diagnosed with early-stage breast cancer could remain event-free up to 5-years following diagnosis [44]. Low event rates and longer event-free intervals among early-stage breast cancer survivors would require a significant investment in large sample sizes and long-term follow-up to quantify the effects of MSE interventions on recurrence and/or mortality in a clinical trial.

With one exception, [35] the isolated association of MSE with recurrence and/or mortality independent of aerobic exercise was not examined. Two articles were cross-sectional, and only three accounted for confounders in analyses [35–37]. There was limited information on variations in the association of MSE with recurrence and/or mortality across various individual demographic (e.g., age, race) or clinical characteristics (e.g., stage at diagnosis). All but one article [34] included samples comprised of predominantly White women. As such, it remains unclear how the association of MSE with recurrence/mortality differs across various subgroups of the population, [45–48] as has been shown when examining the association between aerobic exercise and recurrence/mortality among breast cancer survivors [16, 20, 49–51].

The findings of this study may have several implications for future research and clinical practice. Our review of the measures and methods in the existing literature indicates that future research must measure, categorize, and analyze MSE consistently in a transparent manner to facilitate comparison and synthesis of results across studies. Health care providers may find absolute effects (vs. relative) more useful in informing clinical practice [52]. However, in this review, we found no studies that reported absolute benefits of MSE among breast cancer survivors. Therefore, future research should also consider including estimates of absolute (adjusted) differences in addition to the relative differences (e.g., hazard ratios) in the association of MSE with recurrence/mortality. We also found limited data on the possible variations in the association of MSE with recurrence/mortality based on individual demographic, clinical, and contextual characteristics. This data may be necessary in informing personalized exercise prescriptions as recommended by cancer exercise guidelines [7]. For example, it may be necessary to consider a woman’s stage at diagnosis, surgery, treatment, time since treatment completion, and comorbidities when recommending MSE during survivorship care. There was also limited data on the dose-response relationship of MSE with recurrence/mortality which has been reported for aerobic exercise [18]. Therefore, future research should consider providing data on variations in the association of MSE with recurrence/mortality, and the dose-response relationship between MSE and recurrence/mortality. Current cancer survivorship care guidelines recommend that clinicians discuss exercise as a part of the patient’s survivorship care plan [53, 54]. Our findings may offer clinicians with the necessary evidence to help communicate the long-term benefits (e.g., mortality reductions) of MSE in addition to the short-term benefits (e.g., fatigue, physical function, mental health) [7–10] to breast cancer survivors in clinical settings.

## Conclusions

In summary, limited evidence suggests that MSE may reduce the hazards of recurrence and mortality among breast cancer survivors. However, additional research is warranted to understand the magnitude and variation of these associations. Greater consistency in measurement and analyses within MSE research could help generate findings more readily to inform clinical practice.

## Electronic supplementary material

Below is the link to the electronic supplementary material.

Supplementary Material 1

## Data Availability

Data sharing is not applicable to this article as no datasets were analyzed or generated during the current review. All the studies summarized in this systematic review are clearly identified within the article.

## Declarations

### Disclaimer

The contents and views in this manuscript are those of the authors and should not be construed to represent the views of the National Institutes of Health. Opinions and comments expressed in this paper belong to the authors and do not necessarily reflect those of the U.S. Government, Department of Health and Human Services, National Institutes of Health, the National Institute on Minority Health and Health Disparities, or National Cancer Institute. The review funders had no role in the design of the review, the collection, analysis, or interpretation of the data, the writing of the manuscript, or the decision to submit the manuscript for publication.

## Abbreviations

MSE: Muscle-strengthening exercise
