# Supplementary material for: The associations of muscle-strengthening exercise with recurrence and mortality among breast cancer survivors: a systematic review
Source: Int J Behav Nutr Phys Act. 2024 Sep 10;21:100. doi: 10.1186/s12966-024-01644-0 (PMC11389293; doi:10.1186/s12966-024-01644-0)
Supplement: Supplementary file 1 — Supplementary Material 1 [file 12966_2024_1644_MOESM1_ESM.docx]

**Supplement 1. PRISMA Checklist**

| **Section and Topic** | **Item #** | **Checklist item** | **Location where item is reported** |
| --- | --- | --- | --- |
| **TITLE** | | |  |
| Title | 1 | Identify the report as a systematic review. | Page 1 |
| **ABSTRACT** | | |  |
| Abstract | 2 | See the PRISMA 2020 for Abstracts checklist. | Page 1 |
| **INTRODUCTION** | | |  |
| Rationale | 3 | Describe the rationale for the review in the context of existing knowledge. | Page 2 |
| Objectives | 4 | Provide an explicit statement of the objective(s) or question(s) the review addresses. | Page 2 |
| **METHODS** | | |  |
| Eligibility criteria | 5 | Specify the inclusion and exclusion criteria for the review and how studies were grouped for the syntheses. | Pages 2-3 |
| Information sources | 6 | Specify all databases, registers, websites, organisations, reference lists and other sources searched or consulted to identify studies. Specify the date when each source was last searched or consulted. | Page 2 |
| Search strategy | 7 | Present the full search strategies for all databases, registers and websites, including any filters and limits used. | Supplement 1 |
| Selection process | 8 | Specify the methods used to decide whether a study met the inclusion criteria of the review, including how many reviewers screened each record and each report retrieved, whether they worked independently, and if applicable, details of automation tools used in the process. | Page 3 |
| Data collection process | 9 | Specify the methods used to collect data from reports, including how many reviewers collected data from each report, whether they worked independently, any processes for obtaining or confirming data from study investigators, and if applicable, details of automation tools used in the process. | Page 3 |
| Data items | 10a | List and define all outcomes for which data were sought. Specify whether all results that were compatible with each outcome domain in each study were sought (e.g. for all measures, time points, analyses), and if not, the methods used to decide which results to collect. | Page 3 |
|  | 10b | List and define all other variables for which data were sought (e.g. participant and intervention characteristics, funding sources). Describe any assumptions made about any missing or unclear information. | Page 3 |
| Study risk of bias assessment | 11 | Specify the methods used to assess risk of bias in the included studies, including details of the tool(s) used, how many reviewers assessed each study and whether they worked independently, and if applicable, details of automation tools used in the process. | Page 3 |
| Effect measures | 12 | Specify for each outcome the effect measure(s) (e.g. risk ratio, mean difference) used in the synthesis or presentation of results. | Page 3 |
| Synthesis methods | 13a | Describe the processes used to decide which studies were eligible for each synthesis (e.g. tabulating the study intervention characteristics and comparing against the planned groups for each synthesis (item #5)). | Page 3 |
|  | 13b | Describe any methods required to prepare the data for presentation or synthesis, such as handling of missing summary statistics, or data conversions. | Page 3 |
|  | 13c | Describe any methods used to tabulate or visually display results of individual studies and syntheses. | Page 3 |
|  | 13d | Describe any methods used to synthesize results and provide a rationale for the choice(s). If meta-analysis was performed, describe the model(s), method(s) to identify the presence and extent of statistical heterogeneity, and software package(s) used. | NA |
|  | 13e | Describe any methods used to explore possible causes of heterogeneity among study results (e.g. subgroup analysis, meta-regression). | NA |
|  | 13f | Describe any sensitivity analyses conducted to assess robustness of the synthesized results. | NA |
| Reporting bias assessment | 14 | Describe any methods used to assess risk of bias due to missing results in a synthesis (arising from reporting biases). | Page 3 |
| Certainty assessment | 15 | Describe any methods used to assess certainty (or confidence) in the body of evidence for an outcome. | Pages 3-4 |
| **RESULTS** | | |  |
| Study selection | 16a | Describe the results of the search and selection process, from the number of records identified in the search to the number of studies included in the review, ideally using a flow diagram. | Page 4 |
|  | 16b | Cite studies that might appear to meet the inclusion criteria, but which were excluded, and explain why they were excluded. | Page 4 |
| Study characteristics | 17 | Cite each included study and present its characteristics. | Pages 4-6 |
| Risk of bias in studies | 18 | Present assessments of risk of bias for each included study. | Pages 4, 6 |
| Results of individual studies | 19 | For all outcomes, present, for each study: (a) summary statistics for each group (where appropriate) and (b) an effect estimate and its precision (e.g. confidence/credible interval), ideally using structured tables or plots. | Pages 6-8 |
| Results of syntheses | 20a | For each synthesis, briefly summarise the characteristics and risk of bias among contributing studies. | Page 4-6 |
|  | 20b | Present results of all statistical syntheses conducted. If meta-analysis was done, present for each the summary estimate and its precision (e.g. confidence/credible interval) and measures of statistical heterogeneity. If comparing groups, describe the direction of the effect. | NA |
|  | 20c | Present results of all investigations of possible causes of heterogeneity among study results. | NA |
|  | 20d | Present results of all sensitivity analyses conducted to assess the robustness of the synthesized results. | NA |
| Reporting biases | 21 | Present assessments of risk of bias due to missing results (arising from reporting biases) for each synthesis assessed. | Page 6 |
| Certainty of evidence | 22 | Present assessments of certainty (or confidence) in the body of evidence for each outcome assessed. | Pages 6-8 |
| **DISCUSSION** | | |  |
| Discussion | 23a | Provide a general interpretation of the results in the context of other evidence. | Pages 8-9 |
|  | 23b | Discuss any limitations of the evidence included in the review. | Page 9 |
|  | 23c | Discuss any limitations of the review processes used. | Page 9 |
|  | 23d | Discuss implications of the results for practice, policy, and future research. | Page 9 |
| **OTHER INFORMATION** | | |  |
| Registration and protocol | 24a | Provide registration information for the review, including register name and registration number, or state that the review was not registered. | Page 2 |
|  | 24b | Indicate where the review protocol can be accessed, or state that a protocol was not prepared. | Page 2 |
|  | 24c | Describe and explain any amendments to information provided at registration or in the protocol. | NA |
| Support | 25 | Describe sources of financial or non-financial support for the review, and the role of the funders or sponsors in the review. | Page 10 |
| Competing interests | 26 | Declare any competing interests of review authors. | Page 10 |
| Availability of data, code and other materials | 27 | Report which of the following are publicly available and where they can be found: template data collection forms; data extracted from included studies; data used for all analyses; analytic code; any other materials used in the review. | Page 10 |

*From:* Page MJ, McKenzie JE, Bossuyt PM, Boutron I, Hoffmann TC, Mulrow CD, et al. The PRISMA 2020 statement: an updated guideline for reporting systematic reviews. BMJ 2021;372:n71. doi: 10.1136/bmj.n71

**Supplement 2. Search strategies**

Database: PubMed/MEDLINE
Platform: National Library of Medicine
Date Searched: 9/18/2023

Date Limits: 1/1/2003 – 9/18/2023

Limits: English & Human studies

|  | Concept: | Search Strategy: |
| --- | --- | --- |
| #1 | Breast Cancer | "Breast Neoplasms"[Mesh] OR (("Breast"[Mesh] OR "breast*"[Title/Abstract]) AND ("cancer*"[Title/Abstract] OR "neoplas*"[Title/Abstract] OR "carcinoma*"[Title/Abstract] OR "tumor*"[Title/Abstract] OR "tumour*"[Title/Abstract] OR "malignan*"[Title/Abstract] OR "sarcoma*"[Title/Abstract] OR "lymphoma*"[Title/Abstract])) |
| #2 | Physical Activity | "Exercise"[Mesh] OR "Exercise Movement Techniques"[Mesh] OR "Exercise Therapy"[Mesh] OR "exercise*"[Title/Abstract] OR "Physical Fitness"[Mesh] OR "physical fitness"[Title/Abstract] OR "physical activit*"[Title/Abstract] OR "physical performanc*"[Title/Abstract] OR "Physical Exertion"[Mesh] OR "physical exertion"[Title/Abstract] OR "Physical Endurance"[Mesh] OR "physical endurance"[Title/Abstract:~4] OR "endurance training"[Title/Abstract] OR "physical intensit*"[Title/Abstract] OR "Recreation"[Mesh:NoExp] OR "recreational activit*"[Title/Abstract] OR "Sports"[Mesh] OR "sport*"[Title/Abstract] OR "stretching"[Title/Abstract] OR "Yoga"[Mesh] OR "yoga"[Title/Abstract] OR "Tai Ji"[Mesh] OR "Tai Ji"[Title/Abstract] OR "Tai Chi"[Title/Abstract] OR "muscle strength*"[Title/Abstract] OR "muscle strengthening"[Title/Abstract:~4] OR "resistance training"[Title/Abstract] OR "strength training"[Title/Abstract:~4] OR "muscle strength*"[Title/Abstract] OR "weight training"[Title/Abstract] OR "weightlifting"[Title/Abstract] OR "weight lifting"[Title/Abstract] OR "Walking"[Mesh] OR "walk*"[Title/Abstract] OR "gait"[Title/Abstract] OR "jog"[Title/Abstract] OR "jogging"[Title/Abstract] OR "running"[Title/Abstract] OR "swimming"[Title/Abstract] OR "aerobic*"[Title/Abstract] OR "exertion*"[Title/Abstract] OR "isometric*"[Title/Abstract] OR "energy expenditure*"[Title/Abstract] OR "energy balanc*"[Title/Abstract] OR "energy intake*"[Title/Abstract] OR "circuit*"[Title/Abstract] OR "high intensity interval*"[Title/Abstract] OR "high intensity intermittent*"[Title/Abstract] OR "HIIT"[Title/Abstract] OR "sprint interval*"[Title/Abstract] OR "stair climbing"[Title/Abstract:~3] OR "calisthenic*"[Title/Abstract] OR "cycling*"[Title/Abstract] OR "biking*"[Title/Abstract] OR "Sedentary Behavior"[Mesh] OR "sedentary"[Title/Abstract] OR "physical inactivit*"[Title/Abstract] |
| #3 | Recurrence | "Neoplasm Recurrence, Local"[Mesh] OR "Recurrence"[Mesh] OR "recurren*"[Title/Abstract] OR "reoccurren*"[Title/Abstract] OR "relaps*"[Title/Abstract] OR "Neoplasms, Second Primary"[Mesh] OR "secondary events"[Title/Abstract] OR "second cancer*"[Title/Abstract] OR "second primary cancer*"[Title/Abstract] OR "secondary cancer*"[Title/Abstract] OR "secondary primary cancer*"[Title/Abstract] OR "Disease Progression"[Mesh] OR "progression*"[Title/Abstract] OR "Disease-Free Survival"[Mesh] OR "cancer survivor*"[Title/Abstract] OR "cancer survival"[Title/Abstract:~3] OR "cause death"[Title/Abstract:~3] OR "all cause"[Title/Abstract] OR "fatal*"[Title/Abstract] OR "Mortality"[Mesh] OR "mortality" [Subheading] OR "mortalit*"[Title/Abstract] OR "Quality of Life"[Mesh] OR "quality of life"[Title/Abstract] OR "life quality"[Title/Abstract] OR "HRQOL"[Title/Abstract] |
| #4 | Limits & Filters | ((#1 AND #2 AND #3) NOT ("Animals"[Mesh] NOT ("Animals"[Mesh] AND "Humans"[Mesh]))) NOT (editorial[Publication Type] OR comment[Publication Type] OR "commentary*"[Title/Abstract] OR news[Publication Type] OR letter[Publication Type] OR retracted publication[Publication Type] OR retraction of publication[Publication Type] OR "retraction of publication*"[Title/Abstract] OR "retraction notice"[Title] OR "retracted publication"[Title] OR "Congress"[Publication Type] OR "Consensus Development Conference"[Publication Type] OR "conference abstract*"[Title/Abstract] OR "conference proceeding*"[Title/Abstract] OR "conference paper*"[Title/Abstract] OR "conference review*"[Title/Abstract] OR "symposium*"[Title/Abstract] OR "Case Reports" [Publication Type] OR "case report*"[Title/Abstract] OR "Review" [Publication Type] OR "review"[Title] OR "Systematic Review" [Publication Type] OR "Systematic Reviews as Topic"[Mesh] OR "systematic review"[Title/Abstract] OR "Meta-Analysis" [Publication Type] OR "Meta-Analysis as Topic"[Mesh] OR "meta-analysis "[Title/Abstract] OR "meta-analyses "[Title/Abstract] OR "protocol"[Title] OR "protocols"[Title] OR "Practice Guideline"[Publication Type] OR "guideline*"[Title]) Filters: English, from 2003/1/1 - 2023/9/14 |

Database: Cochrane CENTRAL
Platform: Wiley & Sones
Date Searched: 09/18/2023

Date Limits: 1/1/2003 – 09/18/2023

Limits: English & Human studies

|  | Concept: | Search Strategy: |
| --- | --- | --- |
| #1 | Breast Cancer | ([mh "Breast Neoplasms"] OR (([mh "Breast"] OR breast*) NEAR/4 (cancer* OR neoplas* OR carcinoma* OR tumor* OR tumour* OR malignan* OR sarcoma* OR lymphoma*))):ti,ab,kw |
| #2 | Physical Activity | ([mh "Exercise"] OR [mh "Exercise Movement Techniques"] OR [mh "Exercise Therapy"] OR exercise* OR [mh "Physical Fitness"] OR "physical fitness" OR (physical NEXT activit*) OR (physical NEXT performanc*) OR [mh "Physical Exertion"] OR "physical exertion" OR [mh "Physical Endurance"] OR (physical NEAR/4 endurance) OR "endurance training" OR (physical NEXT intensit*) OR [mh ^"Recreation"] OR (recreational NEXT activit*) OR [mh "Sports"] OR sport* OR stretching OR [mh "Yoga"] OR yoga OR [mh "Tai Ji"] OR "Tai Ji" OR "Tai Chi" OR (muscle NEXT strength*) OR (muscle NEAR/4 strengthening) OR "resistance training" OR (strength NEAR/4 training) OR (muscle NEXT strength*) OR "weight training" OR weightlifting OR "weight lifting" OR [mh "Walking"] OR walk* OR gait OR jog OR jogging OR running OR swimming OR aerobic* OR exertion* OR isometric* OR (energy NEXT expenditure*) OR (energy NEXT balanc*) OR (energy NEXT intake*) OR circuit* OR ("high intensity" NEXT interval*) OR ("high intensity" NEXT intermittent*) OR "HIIT" OR (sprint NEXT interval*) OR (stair* NEAR/3 climbing) OR calisthenic* OR cycling* OR biking* OR [mh "Sedentary Behavior"] OR sedentary OR (physical NEXT inactivit*)):ti,ab,kw |
| #3 | Recurrence | ([mh "Neoplasm Recurrence, Local"] OR [mh "Recurrence"] OR recurren* OR reoccurren* OR relaps* OR [mh "Neoplasms, Second Primary"] OR "secondary events" OR (second NEXT cancer*) OR ("second primary" NEXT cancer*) OR (secondary NEXT cancer*) OR ("secondary primary" NEXT cancer*) OR [mh "Disease Progression"] OR progression* OR [mh "Disease-Free Survival"] OR (cancer NEXT survivor*) OR (cancer NEAR/3 survival) OR (cause NEAR/3 death) OR "all cause" OR fatal* OR [mh "Mortality"] OR mortalit* OR [mh "Quality of Life"] OR "quality of life" OR "life quality" OR "HRQO"):ti,ab,kw |
| #4 |  | #1 AND #2 AND #3 |
| #5 | Limits & Filters | [mh "Editorial"] OR [mh "Comment"] OR commentary* OR [mh "News"] OR [mh "Letter"] OR [mh "Retracted publication"] OR [mh "Retraction of publication"] OR (retraction NEAR/2 publication*) OR [mh "Congress"] OR [mh "Consensus Development Conference"] OR (conference NEXT abstract*) OR (conference NEXT proceeding*) OR (conference NEXT paper*) OR (conference NEXT review*) OR symposium* OR [mh "Case Reports"] OR (case NEXT report*) OR [mh "Review"] OR [mh "Systematic Review"] OR [mh "Systematic Reviews as Topic"] OR "systematic review" OR [mh "Meta-Analysis"] OR [mh "Meta-Analysis as Topic"] OR "meta-analysis" OR "meta-analyses" OR [mh "Practice Guideline"] |
| #6 | Limits & Filters | ("retraction notice" OR "retracted publication" OR review OR protocol OR protocols OR guideline*):ti |
| #7 |  | (#5 OR #6) |
| #8 | Limits & Filters | #4 NOT #7" with Publication Year from 2003 to 2023, with Cochrane Library publication date Between Jan 2003 and Sep 2023, in Trials (Word variations have been searched) |

Database: Embase
Platform: Elsevier
Date Searched: 9/18/2023

Date Limits: 1/1/2003 – 9/18/2023
Limits: English & Human studies

|  | Concept: | Search Strategy: |
| --- | --- | --- |
| #1 | Breast Cancer | 'breast cancer'/exp OR ((breast* NEAR/4 (cancer* OR neoplas* OR carcinoma* OR tumor* OR tumour* OR malignan* OR sarcoma* OR lymphoma*)):ab,ti) |
| #2 | Physical Activity | 'exercise'/exp OR 'kinesiotherapy'/exp OR 'fitness'/exp OR 'endurance'/exp OR 'recreation'/de OR 'sport'/exp OR 'tai chi'/exp OR 'walking'/exp OR 'sedentary lifestyle'/exp OR 'exercise*':ab,ti OR 'physical fitness':ab,ti OR 'physical activit*':ab,ti OR 'physical performanc*':ab,ti OR 'physical exertion':ab,ti OR ((physical NEAR/4 endurance):ab,ti) OR 'endurance training':ab,ti OR 'physical intensit*':ab,ti OR 'recreational activit*':ab,ti OR 'sport*':ab,ti OR 'stretching':ab,ti OR 'yoga':ab,ti OR 'tai ji':ab,ti OR 'tai chi':ab,ti OR ((muscle NEAR/4 strengthening):ab,ti) OR 'resistance training':ab,ti OR ((strength NEAR/4 training):ab,ti) OR 'muscle strength*':ab,ti OR 'weight training':ab,ti OR 'weightlifting':ab,ti OR 'weight lifting':ab,ti OR 'walk*':ab,ti OR 'gait':ab,ti OR 'jog':ab,ti OR 'jogging':ab,ti OR 'running':ab,ti OR 'swimming':ab,ti OR 'aerobic*':ab,ti OR 'exertion*':ab,ti OR 'isometric*':ab,ti OR 'energy expenditure*':ab,ti OR 'energy balanc*':ab,ti OR 'energy intake*':ab,ti OR 'circuit*':ab,ti OR 'high intensity interval*':ab,ti OR 'high intensity intermittent*':ab,ti OR 'hiit':ab,ti OR 'sprint interval*':ab,ti OR ((stair* NEAR/3 climbing):ab,ti) OR 'calisthenic*':ab,ti OR 'cycling*':ab,ti OR 'biking*':ab,ti OR 'sedentary':ab,ti OR 'physical inactivit*':ab,ti |
| #3 | Recurrence | 'tumor recurrence'/exp OR 'recurrent disease'/exp OR 'second primary neoplasm'/exp OR 'disease exacerbation'/exp OR 'disease free survival'/exp OR 'mortality'/mj OR 'quality of life'/exp OR 'recurren*':ab,ti OR 'reoccurren*':ab,ti OR 'relaps*':ab,ti OR 'secondary events':ab,ti OR 'second cancer*':ab,ti OR 'second primary cancer*':ab,ti OR 'secondary cancer*':ab,ti OR 'secondary primary cancer*':ab,ti OR 'progression*':ab,ti OR 'cancer survivor*':ab,ti OR ((cancer NEAR/3 survival):ab,ti) OR ((cause NEAR/3 death):ab,ti) OR 'all cause':ab,ti OR 'fatal*':ab,ti OR 'mortalit*':ab,ti OR 'quality of life':ab,ti OR 'life quality':ab,ti OR 'hrqol':ab,ti |
| #4 | Limits & Filters | #1 AND #2 AND #3 AND ([article]/lim OR [article in press]/lim) AND [english]/lim NOT ([animals]/lim NOT ([animals]/lim AND [humans]/lim)) NOT ('editorial'/exp OR 'letter'/exp OR 'note'/exp OR 'abstract report'/exp OR 'conference paper'/exp OR 'review'/exp OR 'systematic review'/exp OR 'systematic review (topic)'/exp OR 'meta analysis'/exp OR 'meta analysis (topic)'/exp OR 'case report'/exp OR 'practice guideline'/exp OR 'retraction notice'/exp OR 'retraction of publication':ab,ti OR 'retraction notice':ti OR 'retracted publication':ab,ti OR 'systematic review':ab,ti OR 'meta analysis':ab,ti OR 'meta analyses':ab,ti OR 'review':ti OR 'protocol':ti OR 'protocols':ti OR 'guideline*':ti OR 'case report*':ab,ti OR [conference abstract]/lim OR [conference paper]/lim OR [conference review]/lim OR 'symposium*':ab,ti OR 'conference abstract*':ab,ti OR 'conference review*':ab,ti OR 'conference proceeding*':ab,ti OR 'conference paper*':ab,ti) |

Database: CINAHL
Platform: EBSCO*host*
Date Searched: 9/18/2023

Date Limits: 1/1/2003 – 9/18/2023

Limits: English & Human studies

|  | Concept: | Search Strategy: |
| --- | --- | --- |
| #S1 | Breast Cancer | (MH "Breast Neoplasms+") OR TI ( (((MH "Breast+") OR breast*) AND (cancer* OR neoplas* OR carcinoma* OR tumor* OR tumour* OR malignan* OR sarcoma* OR lymphoma*)) ) OR AB ( (((MH "Breast+") OR breast*) AND (cancer* OR neoplas* OR carcinoma* OR tumor* OR tumour* OR malignan* OR sarcoma* OR lymphoma*)) ) |
| #S2 | Physical Activity | ((MH "Exercise+") OR (MH "Therapeutic Exercise+") OR (MH "Physical Fitness+") OR (MH "Exertion+") OR (MH "Physical Endurance+") OR (MH "Recreation") OR (MH "Sports+") OR (MH "Yoga+") OR (MH "Tai Chi") OR (MH "Walking+") OR (MH "Life Style, Sedentary+") ) OR TI ( exercise* OR "physical fitness" OR "physical activit*" OR "physical performanc*" OR "physical exertion" OR (physical W4 endurance) OR "endurance training" OR "physical intensit*" OR "recreational activit*" OR "sport*" OR "stretching" OR "yoga" OR "Tai Ji" OR "Tai Chi" OR "muscle strength*" OR (muscle W4 strengthening") OR "resistance training" OR (strength W4 training") OR "muscle strength*" OR "weight training" OR "weightlifting" OR "weight lifting" OR "walk*" OR "gait" OR "jog" OR "jogging" OR "running" OR "swimming" OR "aerobic*" OR "exertion*" OR "isometric*" OR "energy expenditure*" OR "energy balanc*" OR "energy intake*" OR "circuit*" OR "high intensity interval*" OR "high intensity intermittent*" OR "HIIT" OR "sprint interval*" OR (stair* W3 climbing) OR calisthenic* OR cycling* OR "biking* OR sedentary OR "physical inactivit*" ) OR AB ( exercise* OR "physical fitness" OR "physical activit*" OR "physical performanc*" OR "physical exertion" OR (physical W4 endurance) OR "endurance training" OR "physical intensit*" OR "recreational activit*" OR "sport*" OR "stretching" OR "yoga" OR "Tai Ji" OR "Tai Chi" OR "muscle strength*" OR (muscle W4 strengthening") OR "resistance training" OR (strength W4 training") OR "muscle strength*" OR "weight training" OR "weightlifting" OR "weight lifting" OR "walk*" OR "gait" OR "jog" OR "jogging" OR "running" OR "swimming" OR "aerobic*" OR "exertion*" OR "isometric*" OR "energy expenditure*" OR "energy balanc*" OR "energy intake*" OR "circuit*" OR "high intensity interval*" OR "high intensity intermittent*" OR "HIIT" OR "sprint interval*" OR (stair* W3 climbing) OR calisthenic* OR cycling* OR "biking* OR sedentary OR "physical inactivit*") |
| #S3 | Recurrence | ( (MH "Neoplasm Recurrence, Local") OR (MH "Recurrence+") OR (MH "Neoplasms, Second Primary") OR (MH "Disease Progression+") OR (MH "Disease-Free Survival") OR (MH "Mortality+") OR (MH "Quality of Life+") ) OR TI ( recurren* OR reoccurren* OR relaps* OR "secondary events" OR "second cancer*" OR "second primary cancer*" OR "secondary cancer*"OR "secondary primary cancer*" OR progression* OR "cancer survivor*" OR (cancer N/3 survival) OR (cause N/3 death) OR "all cause" OR fatal* OR mortalit* OR "quality of life" OR "life quality" OR "HRQOL" ) OR AB ( recurren* OR reoccurren* OR relaps* OR "secondary events" OR "second cancer*" OR "second primary cancer*" OR "secondary cancer*"OR "secondary primary cancer*" OR progression* OR "cancer survivor*" OR (cancer N/3 survival) OR (cause N/3 death) OR "all cause" OR fatal* OR mortalit* OR "quality of life" OR "life quality" OR "HRQOL" ) |
| #S4 | Limits & Filters | #S4 NOT ( (((MH "Animals+") OR (MH "Animal Studies") OR (TI "animal model*")) NOT (MH "human")) ) NOT ( (MH "Congresses and Conferences") OR (MH "Edit and Review+") OR (MH "News") OR (MH "Literature Review+") OR (MH "Meta Analysis") OR (MH "Systematic Review") OR (MH "Retracted Publication") OR (MH "Retraction of Publication) OR (MH "Case Studies") OR (MH "Practice Guidelines") ) OR ( "conference abstract*" OR "conference proceeding*" OR "conference paper*" OR "conference review*" OR "symposium*" OR "case report*" OR "systematic review" OR "meta-analysis" OR "meta-analyses" OR "retraction of publication*" ) OR TI ( review OR protocol OR protocols OR "retraction notice" OR "retracted publication" OR guideline* ) Limiters - Publication Year: 2003-2023; Published Date: 20030101-20230931; English Language; Peer Reviewed Expanders - Apply equivalent subjects Search modes - Boolean/Phrase |

Database: PsycInfo
Platform: American Psychological Association
Date Searched: 9/18/2023

Date Limits: 1/1/2003 – 9/18/2023

Limits: English & Human studies

|  | Concept: | Search Strategy: |
| --- | --- | --- |
| #1 | Breast Cancer | Index Terms: {Breast Neoplasms} *OR* Title: ((^55^ OR breast*) AND (cancer* OR neoplas* OR carcinoma* OR tumor* OR tumour* OR malignan* OR sarcoma* OR lymphoma*)) *OR* Abstract: ((^55^ OR breast*) AND (cancer* OR neoplas* OR carcinoma* OR tumor* OR tumour* OR malignan* OR sarcoma* OR lymphoma*)) |
| #2 | Physical Activity | Index Terms: ^56^ OR {Exercise Therapy} OR {Physical Fitness} OR {Physical Endurance} OR {Recreation} OR ^57^ OR {Yoga} OR {Walking} OR {Sedentary Behavior} *OR* Title: exercise* OR "physical fitness" OR "physical activit*" OR "physical performanc*" OR "physical exertion" OR (physical NEAR/4 endurance) OR "endurance training" OR "physical intensit*" OR "recreational activit*" OR "sport*" OR "stretching" OR "yoga" OR "Tai Ji" OR "Tai Chi" OR "muscle strength*" OR (muscle NEAR/4 strengthening") OR "resistance training" OR (strength NEAR/4 training") OR "muscle strength*" OR "weight training" OR "weightlifting" OR "weight lifting" OR "walk*" OR "gait" OR "jog" OR "jogging" OR "running" OR "swimming" OR "aerobic*" OR "exertion*" OR "isometric*" OR "energy expenditure*" OR "energy balanc*" OR "energy intake*" OR "circuit*" OR "high intensity interval*" OR "high intensity intermittent*" OR "HIIT" OR "sprint interval*" OR (stair* NEAR/3 climbing) OR calisthenic* OR cycling* OR "biking* OR sedentary OR "physical inactivit*" *OR* Abstract: exercise* OR "physical fitness" OR "physical activit*" OR "physical performanc*" OR "physical exertion" OR (physical NEAR/4 endurance) OR "endurance training" OR "physical intensit*" OR "recreational activit*" OR "sport*" OR "stretching" OR "yoga" OR "Tai Ji" OR "Tai Chi" OR "muscle strength*" OR (muscle NEAR/4 strengthening") OR "resistance training" OR (strength NEAR/4 training") OR "muscle strength*" OR "weight training" OR "weightlifting" OR "weight lifting" OR "walk*" OR "gait" OR "jog" OR "jogging" OR "running" OR "swimming" OR "aerobic*" OR "exertion*" OR "isometric*" OR "energy expenditure*" OR "energy balanc*" OR "energy intake*" OR "circuit*" OR "high intensity interval*" OR "high intensity intermittent*" OR "HIIT" OR "sprint interval*" OR (stair* NEAR/3 climbing) OR calisthenic* OR cycling* OR "biking* OR sedentary OR "physical inactivit*" |
| #3 | Recurrence | Index Terms: {Relapse (Disorders)} OR {Disease Progression} OR {Death and Dying} OR {Quality of Life} OR Title: recurren* OR Title: reoccurren* OR Title: relaps* OR Title: "secondary events" OR Title: "second cancer*" OR Title: "second primary cancer*" OR Title: "secondary cancer*" OR "secondary primary cancer*" OR Title: progression* OR Title: "cancer survivor*" OR (Title: cancer NEAR/3 survival) OR (Title: cause NEAR/3 death) OR Title: "all cause" OR Title: fatal* OR Title: mortalit* OR Title: "quality of life" OR Title: "life quality" OR Title: "HRQOL" OR Abstract: recurren* OR Abstract: reoccurren* OR Abstract: relaps* OR Abstract: "secondary events" OR Abstract: "second cancer*" OR Abstract: "second primary cancer*" OR Abstract: "secondary cancer*" OR "secondary primary cancer*" OR Abstract: progression* OR Abstract: "cancer survivor*" OR (Abstract: cancer NEAR/3 survival) OR (Abstract: cause NEAR/3 death) OR Abstract: "all cause" OR Abstract: fatal* OR Abstract: mortalit* OR Abstract: "quality of life" OR Abstract: "life quality" OR Abstract: "HRQOL" |
| #4 | Limits & Filters | ((IndexTermsFilt: ("Breast Neoplasms")) OR (Any Field: TitleFilt: ((("Breast" OR breast*)) AND TitleFilt: ((cancer* OR neoplas* OR carcinoma* OR tumor* OR tumour* OR malignan* OR sarcoma* OR lymphoma*)))) OR (Any Field: AbstractFilt: ((("Breast" OR breast*)) AND AbstractFilt: ((cancer* OR neoplas* OR carcinoma* OR tumor* OR tumour* OR malignan* OR sarcoma* OR lymphoma*))))) AND ((IndexTermsFilt: ("Exercise") OR IndexTermsFilt: ("Exercise Therapy") OR IndexTermsFilt: ("Physical Fitness") OR IndexTermsFilt: ("Physical Endurance") OR IndexTermsFilt: ("Recreation") OR IndexTermsFilt: ("Sports") OR IndexTermsFilt: ("Yoga") OR IndexTermsFilt: ("Walking") OR IndexTermsFilt: ("Sedentary Behavior")) OR title: (exercise* OR "physical fitness" OR "physical activit*" OR "physical performanc*" OR "physical exertion" OR (physical NEAR/4 endurance) OR "endurance training" OR "physical intensit*" OR "recreational activit*" OR "sport*" OR "stretching" OR "yoga" OR "Tai Ji" OR "Tai Chi" OR "muscle strength*" OR (muscle NEAR/4 strengthening ") OR " resistance training " OR (strength NEAR/4 training") OR "muscle strength*" OR "weight training" OR "weightlifting" OR "weight lifting" OR "walk*" OR "gait" OR "jog" OR "jogging" OR "running" OR "swimming" OR "aerobic*" OR "exertion*" OR "isometric*" OR "energy expenditure*" OR "energy balanc*" OR "energy intake*" OR "circuit*" OR "high intensity interval*" OR "high intensity intermittent*" OR "HIIT" OR "sprint interval*" OR (stair* NEAR/3 climbing) OR calisthenic* OR cycling* OR "biking* OR sedentary OR " physical inactivit* ") OR Abstract:(exercise* OR " physical fitness " OR " physical activit* " OR " physical performanc* " OR " physical exertion " OR (physical NEAR/4 endurance) OR " endurance training " OR " physical intensit* " OR " recreational activit* " OR " sport* " OR " stretching " OR " yoga " OR " Tai Ji " OR " Tai Chi " OR " muscle strength* " OR (muscle NEAR/4 strengthening") OR Any Field: "resistance training" OR (strength NEAR/4 training ") OR " muscle strength* " OR " weight training " OR " weightlifting " OR " weight lifting " OR " walk* " OR " gait " OR " jog " OR " jogging " OR " running " OR " swimming " OR " aerobic* " OR " exertion* " OR " isometric* " OR " energy expenditure* " OR " energy balanc* " OR " energy intake* " OR " circuit* " OR " high intensity interval* " OR " high intensity intermittent* " OR " HIIT " OR " sprint interval* " OR (stair* NEAR/3 climbing) OR calisthenic* OR cycling* OR " biking* OR sedentary OR "physical inactivit*")) AND ((IndexTermsFilt: ("Relapse (Disorders)") OR IndexTermsFilt: ("Disease Progression") OR IndexTermsFilt: ("Death and Dying") OR IndexTermsFilt: ("Quality of Life")) OR (title: (recurren*) OR title: (reoccurren*) OR title: (relaps*) OR title: ("secondary events") OR title: ("second cancer*") OR title: ("second primary cancer*") OR title: ("secondary cancer*" OR "secondary primary cancer*") OR title: (progression*) OR title: ("cancer survivor*") OR (title: (cancer NEAR/3 survival)) OR (title: (cause NEAR/3 death)) OR title: ("all cause") OR title: (fatal*) OR title: (mortalit*) OR title: ("quality of life") OR title: ("life quality") OR title: ("HRQOL")) OR (abstract: (recurren*) OR abstract: (reoccurren*) OR abstract: (relaps*) OR abstract: ("secondary events") OR abstract: ("second cancer*") OR abstract: ("second primary cancer*") OR abstract: ("secondary cancer*" OR "secondary primary cancer*") OR abstract: (progression*) OR abstract: ("cancer survivor*") OR (abstract: (cancer NEAR/3 survival)) OR (abstract: (cause NEAR/3 death)) OR abstract: ("all cause") OR abstract: (fatal*) OR abstract: (mortalit*) OR abstract: ("quality of life") OR abstract: ("life quality") OR abstract: ("HRQOL"))) AND Language: English AND NOT Population Group: Animal NOT Document Type: Abstract Collection OR Column/Opinion OR Comment/Reply OR Dissertation OR Editorial OR Letter OR Retraction OR Review-Book AND Peer-Reviewed Journals only AND Year: 2003 To 2023 |

Database: Web of Science (Core Collection)
Platform: Clarivate Analytics
Date Searched: 9/18/2023

Date Limits: 1/1/2003 – 9/18/2023

Limits: English & Human studies

|  | Concept: | Search Strategy: |
| --- | --- | --- |
| #1 | Breast Cancer | TS=(breast* AND (cancer* OR neoplas* OR carcinoma* OR tumor* OR tumour* OR malignan* OR sarcoma* OR lymphoma*)) |
| #2 | Physical Activity | TS=(exercise* OR "physical fitness" OR "physical activit*" OR "physical performanc*" OR "physical exertion" OR (physical* NEAR/4 endurance*) OR "endurance training" OR "physical intensit*" OR "recreational activit*" OR sport* OR stretching OR yoga OR "Tai Ji" OR "Tai Chi" OR "muscle strength*" OR (muscle NEAR/4 strengthening) OR "resistance training" OR (strength NEAR/4 training) OR "muscle strength*" OR "weight training" OR "weightlifting" OR "weight lifting" OR walk*OR gait OR jog OR jogging OR running OR swimming OR aerobic* OR exertion* OR isometric* OR "energy expenditure*" OR "energy balanc*" OR "energy intake*" OR circuit* OR "high intensity interval*" OR "high intensity intermittent*" OR HIIT OR "sprint interval*" OR (stair NEAR/3 climbing) OR calisthenic* OR cycling* OR biking* OR sedentary OR "physical inactivit*") |
| #3 | Recurrence | TS=(recurren* OR reoccurren* OR relaps* OR "secondary events" OR "second cancer*" OR "second primary cancer*" OR "secondary cancer*" OR "secondary primary cancer*" OR "progression*" OR "cancer survivor*" OR (cancer NEAR/3 survival) OR (cause NEAR/3 death) OR "all cause" OR fatal* OR mortalit* OR "quality of life" OR "life quality" OR HRQOL) |
| #4 | Limits & Filters | #3 AND #2 AND #1 NOT TS=(Commentary OR "retraction of publication*" OR "conference abstract*" OR "conference proceeding*" OR "conference paper*" OR "conference review*" OR "symposium*" OR "case report*" OR "systematic review" OR "meta-analysis" OR "meta-analyses")) NOT TI=(review OR "retraction notice" OR "retracted publication" OR protocol OR protocols OR guideline*) and Preprint Citation Index (Exclude – Database) and Clinical Trial or Other or Review Article or Clinical Trial or Abstract or Meeting or Dissertation Thesis or Data Set or Editorial Material or Book or Unspecified or Patent or Case Report or Data Study or Letter or News or Correction or Reference Material or Retracted Publication or Data Paper or Biography (Exclude – Document Types) and Animals or Disease Models Animal or Mice Inbred Balb C or Mice Nude or Mice or Cell Movement (Exclude – MeSH Headings) and English (Languages) and Web of Science Core Collection (Database) |

Database: Scopus
Platform: Elsevier

Date Searched: 9/18/2023

Date Limits: 1/1/2003 – 9/18/2023

Limits: English & Human studies

|  | Concept: | Search Strategy: |
| --- | --- | --- |
| #1 | Breast Cancer | TITLE-ABS-KEY(breast* AND (cancer* OR neoplas* OR carcinoma* OR tumor* OR tumour* OR malignan* OR sarcoma* OR lymphoma*)) |
| #2 | Physical Activity | TITLE-ABS-KEY(exercise* OR "physical fitness" OR "physical activit*" OR "physical performanc*" OR "physical exertion" OR (physical* W/4 endurance*) OR "endurance training" OR "physical intensit*" OR "recreational activit*" OR sport* OR stretching OR yoga OR "Tai Ji" OR "Tai Chi" OR "muscle strength*" OR (muscle W/4 strengthening) OR "resistance training" OR (strength W/4 training) OR "muscle strength*" OR "weight training" OR "weightlifting" OR "weight lifting" OR walk*OR gait OR jog OR jogging OR running OR swimming OR aerobic* OR exertion* OR isometric* OR "energy expenditure*" OR "energy balanc*" OR "energy intake*" OR circuit* OR "high intensity interval*" OR "high intensity intermittent*" OR HIIT OR "sprint interval*" OR (stair W/3 climbing) OR calisthenic* OR cycling* OR biking* OR sedentary OR "physical inactivit*") |
| #3 | Recurrence | TITLE-ABS-KEY(recurren* OR reoccurren* OR relaps* OR "secondary events" OR "second cancer*" OR "second primary cancer*" OR "secondary cancer*" OR "secondary primary cancer*" OR "progression*" OR "cancer survivor*" OR (cancer W/3 survival) OR (cause W/3 death) OR "all cause" OR fatal* OR mortalit* OR "quality of life" OR "life quality" OR HRQOL) |
| #4 | Limits & Filters | (TITLE-ABS-KEY((breast* AND (cancer* OR neoplas* OR carcinoma* OR tumor* OR tumour* OR malignan* OR sarcoma* OR lymphoma*)))) AND (TITLE-ABS-KEY(exercise* OR "physical fitness" OR "physical activit*" OR "physical performanc*" OR "physical exertion" OR (physical* W/4 endurance*) OR "endurance training" OR "physical intensit*" OR "recreational activit*" OR sport* OR stretching OR yoga OR "Tai Ji" OR "Tai Chi" OR "muscle strength*" OR (muscle W/4 strengthening) OR "resistance training" OR (strength W/4 training) OR "muscle strength*" OR "weight training" OR "weightlifting" OR "weight lifting" OR walk*OR gait OR jog OR jogging OR running OR swimming OR aerobic* OR exertion* OR isometric* OR "energy expenditure*" OR "energy balanc*" OR "energy intake*" OR circuit* OR "high intensity interval*" OR "high intensity intermittent*" OR HIIT OR "sprint interval*" OR (stair W/3 climbing) OR calisthenic* OR cycling* OR biking* OR sedentary OR "physical inactivit*")) AND (TITLE-ABS-KEY(recurren* OR reoccurren* OR relaps* OR "secondary events" OR "second cancer*" OR "second primary cancer*" OR "secondary cancer*" OR "secondary primary cancer*" OR "progression*" OR "cancer survivor*" OR (cancer W/3 survival) OR (cause W/3 death) OR "all cause" OR fatal* OR mortalit* OR "quality of life" OR "life quality" OR HRQOL)) AND NOT INDEXTERMS (animal OR animals) AND NOT ((TITLE-ABS-KEY(Commentary OR "retraction of publication*" OR "conference abstract*" OR "conference proceeding*" OR "conference paper*" OR "conference review*" OR "symposium*" OR "case report*" OR "systematic review" OR "meta-analysis" OR "meta-analyses") OR TITLE(review OR "retraction notice" OR "retracted publication" OR protocol OR protocols OR guideline*))) AND PUBYEAR > 2002 AND PUBYEAR < 2024 AND (EXCLUDE (DOCTYPE,"re") OR EXCLUDE (DOCTYPE,"ch") OR EXCLUDE (DOCTYPE,"no") OR EXCLUDE (DOCTYPE,"ed") OR EXCLUDE (DOCTYPE,"le") OR EXCLUDE (DOCTYPE,"cp") OR EXCLUDE (DOCTYPE,"bk") OR EXCLUDE (DOCTYPE,"cr")) |
